# Was the Chlamydial Adaptative Strategy to Tryptophan Starvation an Early Determinant of Plastid Endosymbiosis?

**DOI:** 10.3389/fcimb.2016.00067

**Published:** 2016-06-22

**Authors:** Ugo Cenci, Mathieu Ducatez, Derifa Kadouche, Christophe Colleoni, Steven G. Ball

**Affiliations:** Unité de Glycobiologie Structurale et Fonctionnelle, UMR8576 Centre National de la Recherche Scientifique, Université des Sciences et Technologies de Lille, Villeneuve d'AscqFrance

**Keywords:** plastid, endosymbiosis, tryptophan metabolism, Chlamydiales, Photosynthesis

## Abstract

Chlamydiales were recently proposed to have sheltered the future cyanobacterial ancestor of plastids in a common inclusion. The intracellular pathogens are thought to have donated those critical transporters that triggered the efflux of photosynthetic carbon and the consequent onset of symbiosis. Chlamydiales are also suspected to have encoded glycogen metabolism TTS (Type Three Secretion) effectors responsible for photosynthetic carbon assimilation in the eukaryotic cytosol. We now review the reasons underlying other chlamydial lateral gene transfers evidenced in the descendants of plastid endosymbiosis. In particular we show that half of the genes encoding enzymes of tryptophan synthesis in Archaeplastida are of chlamydial origin. Tryptophan concentration is an essential cue triggering two alternative modes of replication in Chlamydiales. In addition, sophisticated tryptophan starvation mechanisms are known to act as antibacterial defenses in animal hosts. We propose that Chlamydiales have donated their tryptophan operon to the emerging plastid to ensure increased synthesis of tryptophan by the plastid ancestor. This would have allowed massive expression of the tryptophan rich chlamydial transporters responsible for symbiosis. It would also have allowed possible export of this valuable amino-acid in the inclusion of the tryptophan hungry pathogens. Free-living single cell cyanobacteria are devoid of proteins able to transport this amino-acid. We therefore investigated the phylogeny of the Tyr/Trp transporters homologous to *E. coli* TyrP/Mre and found yet another LGT from Chlamydiales to Archaeplastida thereby considerably strengthening our proposal.

## A growing case for the direct involvement of Chlamydiales in plastid endosymbiosis

The case for a direct involvement of Chlamydiales in plastid endosymbiosis started with the discovery of an unexpectedly high number of LGTs (Lateral Gene Transfers) uniting the *C. trachomatis* genes to plants (Stephens et al., 1998). Yet, no extant plants are known to be infected by Chlamydiales. Stephens et al. (1998) correctly concluded that these LGTs were probably very ancient and could be dated back to a time when the protist ancestor of plants displayed exposed membranes, and therefore were infected by related pathogens. As the databases increased in size, the initial observations by Stephens et al. (1998) were strengthened by several independent studies that all pointed to a selective enrichment of Archaeplastida in LGTs from Chlamydiales (Brinkman et al., 2002; Huang and Gogarten, 2007; Becker et al., 2008; Moustafa et al., 2008; Collingro et al., 2011; Ball et al., 2013). Archaeplastida are defined by the three major eukaryotic lineages that diversified after primary plastid endosymbiosis (Rhodophyceae: red algae; Chloroplastida: green algae and land-plants and Glaucophyta: glaucophytes). Because nearly half of the LGTs detected were shared between several of the three distinct Archaeplastida lineages, it was concluded that these events could be dated back to their common ancestors, over a billion years ago, back to the time of plastid endosymbiosis (Huang and Gogarten, 2007; Becker et al., 2008; Moustafa et al., 2008; Collingro et al., 2011; Ball et al., 2013). Plastid endosymbiosis consists of a process by which a eukaryote phagotroph internalized a cyanobacterial ancestor (the cyanobiont) and established a symbiotic link consisting of photosynthetic carbon export from the cyanobiont to the host cytosol (McFadden, 2014). Huang and Gogarten (2007) were the first to propose that this chlamydial phylogenetic signal could be explained if the pathogens were persistent and took an active part in the process of metabolic integration of the evolving organelle. Independently from these phylogenetic studies, metabolic networks that operated in the common ancestor of Archaeplastida were reconstructed through comparison of the accessible red and green algae genome sequences (Deschamps et al., 2008). The primordial carbon flux that united the two disconnected host and cyanobacterial enzyme networks was, from such studies, proposed to consist of the export of the bacterial specific glycosyl-nucleotide ADP-Glc (adenosine diphosphate-glucose) and its incorporation into the host glycogen pools. However, a glycogen synthase utilizing a substrate absent from eukaryotes was unlikely to have been encoded by the host prior to endosymbiosis. From the phylogeny of extant enzymes, it was proposed that this glycogen synthase was a Chlamydiales effector secreted by the TTS (type three secretion system) into the host cytosol. This prediction was verified both by a *semi-in vitro* system using a heterologous *Shigella flexnerii* system and by *in vivo* immunolocalization (Ball et al., 2013; Lu et al., 2013). This lead to propose that three rather than two genomes were united in the coding of functions involved in symbiosis establishment. The “Ménage-à-trois” (MAT) hypothesis explained both, the phylogenetic signal found in Archaeplastida, and the molecular nature of the carbon flux involved (Ball et al., 2013). In its first version, the MAT proposed that the cyanobiont escaped from a phagocytic vacuole to the cytosol of a host that had been infected previously by a Chlamydiales (Ball et al., 2013). Following the publication of the first glaucophyte genome description (Price et al., 2012), Facchinelli et al. (2013a) reported that UhpC, a chlamydial Glucose-6-P transporter was likely derived from the true ancestral carbon translocator that exported photosynthetic carbon from the cyanobiont. To accommodate all results, Facchinelli et al. (2013b) proposed that the cyanobacterium and the chlamydial pathogen/symbiont were located in the same inclusion. Glucose-6-P was suggested to have been exported from the cyanobiont by UhpC into the inclusion lumen where it drove glycogen synthesis. Chlamydial-driven glycogen synthesis was indeed recently shown to occur in the *C. trachomatis* inclusion (Gehre et al., 2016). In this case glycogen synthesis was sustained independently of UhpC and in the absence of cyanobiont by the influx of carbon from the host cytosol. Furthermore, the excess carbon available in the inclusion in this version of the MAT was proposed to have been exported to the cytosol in the form of ADP-Glc, thanks to the presence of a nucleotide sugar translocator (NST) on the inclusion membrane. The latter had been previously suspected to have played a major role in plastid endosymbiosis (Colleoni et al., 2010; reviewed in Ball et al., 2015). Here again, very recent studies have proven that NSTs do provide substrate for glycogen synthesis in the inclusion lumen of Chlamydiaceae (Gehre et al., 2016). This modified version of the MAT displays many advantages by comparison to the initial proposal. First, it explains through a single co-infection the simultaneous presence of all partners of the tripartite symbiosis. Second, and most importantly, it proposes a mechanism by which the cyanobiont would have been sheltered from the host antibacterial defense mechanisms, since the inclusion is tailored to achieve this. Third, because Simkaniaceae and Parachlamydiaceae are known to contain functional type four secretion systems (Collingro et al., 2011), the confined inclusion environment would have facilitated conjugative transfer of Chlamydiales genes to the cyanobiont. This, in turn, would have allowed the expression of hydrophobic chlamydial transporters, which are unlikely to define TTS cargo, on the cyanobiont inner membrane at the very onset of plastid endosymbiosis. This early transfer of key chlamydial transporter genes was indeed required to initiate symbiosis. Among the chlamydial transporters evidenced on the plastid inner membranes of all Archaeplastida (green and red algae and glaucophytes) is the ATP import protein, the hallmark of “energy parasitism” of many intracellular bacteria and of all Chlamydiales. It is known that free living mutant cyanobacteria defective for the accumulation of glycogen stores die in darkness, possibly as a consequence of ATP starvation (Gründel et al., 2012; Xu et al., 2013). Cyanobacteria exporting their photosynthetic carbon are expected to mimic such glycogen defective mutants. Simultaneous transfer of both the UhpC protein and the ATP carrier would have made carbon efflux possible and viable. This would have been greatly facilitated through such conjugative transfers. Weak evidence supporting such transfers has indeed been previously produced by Everett et al. (1999). Despite its appeal the phylogenetic foundations of the MAT have been recently questioned because of uncertainties evidenced in some of the single gene phylogenies sustaining it (Domman et al., 2015). However, these uncertainties can be confidently resolved by the use of straightforward biochemical reasoning (Ball et al., 2016), thereby confirming the suspected role of Chlamydiales in plastid endosymbiosis.

## Do lateral gene transfers from Chlamydiales to Archaeplastida bear functional relevance?

The transfer of key chlamydial transporter genes such as UhpC responsible for initiating carbon efflux from the cyanobiont and the ATP carrier which obviates the ensuing negative consequences on cyanobacterial physiology, bear very clear and strong functional relevance (Ball et al., 2015). Equally crucial was the presence of the glycogen metabolism TTS effectors (GlgA and GlgX) responsible for connecting supply and demand of photosynthetic carbon in the host cytosol (Ball et al., 2013). In both cases, Chlamydiales provided key proteins previously absent respectively from the cyanobiont inner membrane or from the host cytosol. However, all chlamydial LGTs evidenced in Archaeplastida do not encode critical proteins suspected to have been previously absent from the cyanobiont. In fact, a majority of the ~50 chlamydial LGTs found in Archaeplastida have replaced the corresponding cyanobacterial genes (Ball et al., 2013). At first glance, this could bear weak or no functional relevance. The chlamydial gene may, indeed, have been transferred by chance faster than the native cyanobacterial gene.

By contrast to these chance replacements, it is also possible that chlamydial versions may have been favored because of strong functional selection. In this respect, thinking about biotic host-pathogen interactions may lead to additional insights to our understanding of chlamydial LGTs in specific pathways. From considerations developed previously, the reasons underlying the selection of the glycogen metabolism effectors are obvious. We will address here the possible reasons underlying the selection of chlamydia LGTs affecting tryptophan metabolism. Tryptophan synthesis is, by far, the most energy consuming of all amino-acid synthesis pathways (Akashi and Gojobori, 2002). Tryptophan availability, as a consequence, constitutes the most important environmental cue triggering the switch between the virulent and persistent mode of pathogen replication (reviewed by Abdelrahman and Belland, 2005; Bonner et al., 2014). In mammalian cells, both host and pathogens have respectively evolved a number of biotic interactions based on tryptophan starvation and starvation evasion respectively (Wood et al., 2004; Ouellette et al., 2006; reviewed by Bonner et al., 2014). Proteins involved in each of these two distinct modes of chlamydia replication are clearly under Trp content enrichment (Trp-up) selection or on the contrary under Trp-down selection in all Chlamydiales proteomes (Lo et al., 2012; Bonner et al., 2014). In particular, transporters such as the aforementioned ATP carrier, the UhpC and the TyrP/Mre (tryptophan and tyrosine) transporters are notably enriched in tryptophan residues, and are considered as paradigm genes involved in the virulent mode of chlamydial replication. Tryptophan is synthesized from chorismic acid by five distinct enzymatic steps involving seven enzymes subunits. Among all Chlamydiales, *Simkania negevensis* is the only organism that contains the full suite of aromatic amino acid metabolism genes including tryptophan synthesis from chorismic acid (Collingro et al., 2011; Bonner et al., 2014). The seven *Trp* genes are organized in an operon that in addition contains the TrpR repressor gene together with an attenuator sequence and the *AroA* locus that encodes the enzyme catalyzing the first committed step of the shikimate pathway that leads to chorismate. This operon has been recently transferred, probably by conjugation, to *Coxiella burnetii*, another intracellular bacterium, where it was inactivated by mutation leading to the presence of pseudogenes (Xie et al., 2003; Bonner et al., 2014). Chlamydiaceae, on the other hand, are either lacking anthranilate synthase only, or a number of additional enzymes of the pathway (Collingro et al., 2011; Bonner et al., 2014; Subtil et al., 2014). Unlike Simkaniaceae, the Chlamydiaceae have indeed resorted to bypass the classical pathway by synthesizing anthranilate from other sources than chorismate, such as the host metabolite kynurenine, or the indole produced by other bacteria, thereby illustrating one of the many adaptative responses of these pathogens to the diverse host-induced tryptophan starvation defense mechanisms (Bonner et al., 2014). Other Chlamydiales, with the exception of Simkaniaceae, have lost the ability to synthesize aromatic amino-acid and therefore rely entirely on their hosts for tryptophan supply (Collingro et al., 2011; Subtil et al., 2014).

## Four out of eight Trp synthesis and export genes are of chlamydial origin in Archaeplastida

**Figure 1:**
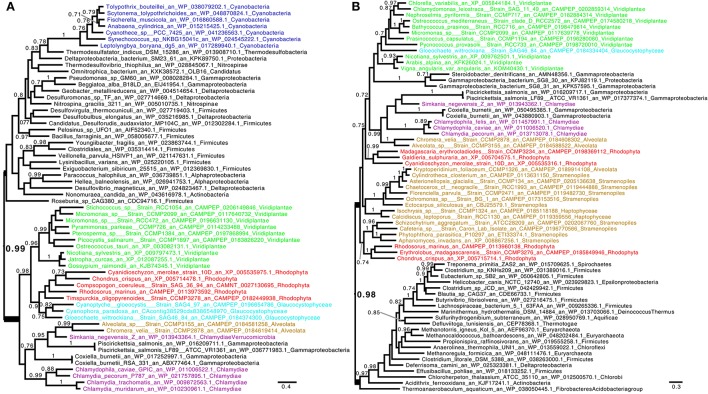
**Consensus tree obtained with Phylobayes 4.1 with Bayesian posterior probabilities mapped onto the nodes of (A) phosphoribosylanthranilate isomerase (TrpF) and (B) anthranilate phosphoribosyltransferase (TrpD)**. Groups of interest are highlighted in purple (Chlamydiales), green (green algae and plants), red (red algae), cyan (Glaucophyta), and blue (Cyanobacteria). Lineages putatively derived from secondary endosymbiosis of Archaeplastida are displayed in brown. Bayesian posterior probabilities values (*PP*) higher than 0.7 are indicated onto the branches. The scale bars indicate the inferred number of amino acid substitutions per site. The trees were manually rooted for convenience of display. The nodes uniting Archaeplastida and their derived lineages to Chlamydiales by LGT are highlighted in bold. The Phosphoribosylanthranilate isomerase tree **(A)** shows a group with robust support (*PP* = 0.99) composed of Chlamydiales, all Archaeplastida, Alveolata, and the two intracellular γ-proteobacteria pathogens *Coxiella* and *Piscirickettsia*. Bonner et al. (2014) have proposed that Coxiella received its Tryptophan operon through LGT from Simkaniaceae (Chlamydiales) in a common intracellular environment (see text). **(B)** shows that the three Archaeplastida lineages are united through LGT together with a complex pattern of secondary endosymbiosis lineages (*PP* = 0.98). Here again intracellular γ-proteobacteria are recovered for the same reasons. Sequences used in the trees were retrieved by homology searches with BLAST against sequences of interest in a database composed of nr, MMETSP (Keeling et al., 2014), and genomes of interest. Sequences with an *E* < 1e-10 were selected and aligned using MAFFT (Katoh and Standley, 2013). We used BMGE (Criscuolo and Gribaldo, 2010) with a block size of four and the BLOSUM30 similarity matrix for block selection. We generated preliminary trees using Fasttree (Price et al., 2010). “Dereplication,” using TreeTrimmer (Maruyama et al., 2013), was applied to supported monophyletic clades in order to reduce sequence redundancy. The final set of sequences were selected manually. Finally, the sequences were re-aligned with MUSCLE (Edgar, 2004), block selection was carried out using BMGE with the same setting, and trees were generated using Phylobayes 4.1 (Lartillot et al., 2009) under the CAT+GTR model (Lartillot and Philippe, 2004). The two chains were stopped when convergence was reached (maxdiff < 0.1) after at least 300 cycles and a burn-in different for each alignment when equilibrium was reached between two chains.

**Figure 2:**
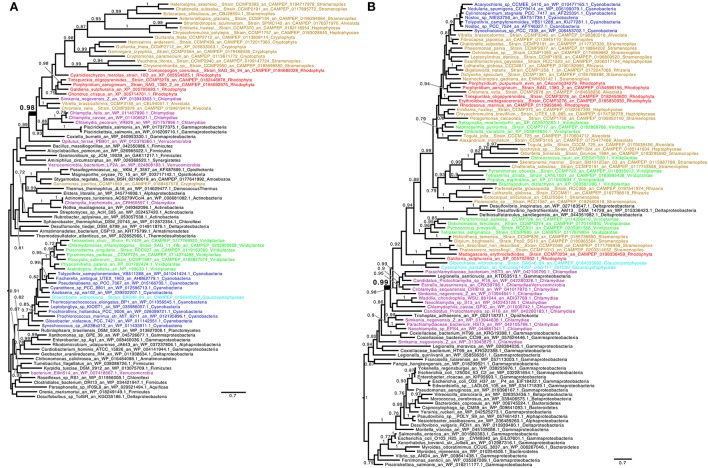
**Consensus tree obtained with Phylobayes 4.1 with Bayesian posterior probabilities mapped onto the nodes of (A) indole-3-glycero phosphate synthase (TrpC) and (B) tyrosine/tryptophan permease (TyrP/Mtr)**. Bayesian posterior probabilities values (*PP*) higher than 0.7 are indicated onto the branches. The scale bars indicate the inferred number of amino acid substitutions per site. The trees were manually rooted for convenience of display. Groups of interest are highlighted in purple (Chlamydiales), green (green algae and plants), red (red algae), cyan (Glaucophyta), and blue (Cyanobacteria). Lineages putatively derived from secondary endosymbiosis of Archaeplastida are displayed in brown. The nodes uniting Archaeplastida and their derived lineages to Chlamydiales by LGT are highlighted in bold. Methods have been detailed in the legend of Figure 1. **(A)** The red algae and Chlamydiales are shown to be united through LGT (highlighted in bold, *PP* = 0.98), however the Green algae and plants and the Glaucophyta have acquired the gene from Cyanobacteria (*PP* = 1). *Coxiella* has been previously proposed to have received their genes from Simkaniaceae (Bonner et al., 2014). **(B)** The putative tyrosine/tryptophan transporter phylogeny displayed has been rooted within γ-proteobacteria for reasons discussed in the text. The node uniting Archaeplastida, secondary endosymbiosis lineages derived from them, Cyanobacteria and a few δ-proteobacteria is highlighted in bold (*PP* = 0.99). The transporter has experienced repeated exchanges in its gene history. Such multiple exchanges between intracellular bacteria are displayed. The root proposed supports a scenario where the gene originated from deeply rooted γ-proteobacteria which diversified in free living, facultative intracellular and obligatory intracellular lineages. In a common intracellular environment the gene was transferred by LGT to Chlamydiales and further exchanged on multiple occasions with intracellular γ-proteobacteria. The Chlamydiales have donated TyrP to Archaeplastida which have donated this to a restricted number of δ-proteobacteria and Nostocales. Only 2 Cyanobacterial lineages not related to Nostocales have been detected and are displayed. For convenience of display the total numbers of representatives of both cyanobacteria and γ-proteobacteria, respectively, of 29 and 692 sequences with at least a different genus have been reduced (see Methods in Figure 1). No TyrP/Mtr transporter have ever been studied in plants. The distantly related PheP *E. coli* translocator was only very recently identified in Petunia and shown to indeed define the major phenylalanine transporter of plant plastids (Widhalm et al., 2015). Neither PheP nor TyrP/Mtr could be recovered from documented plastid proteomes. Nevertheless, TyrP/Mtr green alga and plant sequences closest to the *E. coli* sequence do carry candidate transit peptide sequences. TyrP/Mtr is the only transporter known qualifying as a candidate Trp/Phe transporter in Chlamydiaceae.

One previous study has documented the phylogeny of the Trp synthesis genes of Archaeplastida (Reyes-Prieto and Moustafa, 2012). This study showed that the anthranilate synthase alpha subunit (TrpE), the anthranilate phosphoribosyltransferase (TrpD) and phosphoribosylanthranilate isomerase (TrpF) defining the first, second and third biosynthetic steps were all of non-cyanobacterial origin. Three previous studies had documented either TrpD or TrpF or both as LGTs from Chlamydiales (Huang and Gogarten, 2007; Moustafa et al., 2008; Ball et al., 2013). Because all previous studies did not lead to identical conclusions, we readdressed the phylogenies of these enzymes with more extensive databases. We confirm (Supplementary Figure 1) the results published by Reyes-Prieto and Moustafa (2012) concerning TrpE and also conclude that this gene experienced a clear LGT from Planctomycetes to Archaeplastida. On the other hand, we present in Figures 1A,B the phylogenetic trees corresponding to TrpE and TrpF respectively. Both of these are here confirmed as chlamydial LGTs. We have reproduced the phylogenies of TrpA, TrpB, and TrpG coding, respectively, the two tryptophan synthase α and β subunits and the anthranilate synthase β subunit and conclude that they all display very clear-cut cyanobacterial ancestry, as previously demonstrated (Reyes-Prieto and Moustafa, 2012). However, we now present the phylogeny of TrpC which, possibly because of its complex pattern, was not previously reported. TrpC encodes indole-3-glycerol phosphate synthase the fourth and penultimate step of tryptophan biosynthesis. While complex, this phylogeny shows that the Archaeplastida are here distinctively polyphyletic (Figure 2A). Green alga, glaucophyte, and plant sequences cluster clearly with cyanobacteria while, as shown in Figure 2A, red algae have experienced yet another previously undetected but well supported chlamydial LGT.

Hence on a total of seven protein subunits, three are entirely cyanobacterial in all Archaeplastida, two are entirely chlamydial, one is entirely planctomycetal, and one is partly chlamydial (red algae) and partly cyanobacterial (glaucophytes and green algae). The Planctomycete LGT of TrpE bears strong functional significance since the alpha subunit of anthranilate synthase exerts, by far, the strongest and most significant control on the flux to tryptophan. It is quite striking that all cyanobacteria in the TrpE phylogeny (Reyes-Prieto and Moustafa, 2012; and our results) behave as an uninterrupted large monophyletic group displaying congruence with 16S rRNA phylogeny. This proves that unlike other genes of the Trp operon, this particular cyanobacterial sequence has never been exchanged with other bacteria possibly because of the high selection on its finely tuned regulation. Hence a planctomycete LGT to cyanobacteria prior to endosymbiosis is unlikely to explain the presence of this sequence in Archaeplastida metabolism. We cannot distinguish however a planctomycete LGT that selectively happened upon transfer of the coding capacity to the nucleus from an LGT that took place on the evolving plastid DNA. In this respect it is worth stressing that Planctomycetes Verrumicrobia and Chlamydiales are members of the same so-called PVC supergroup. Because we only have one Chlamydiales (*Simkania negevensis*) sequence encoding a catalytic anthranilate synthase subunit, it is possible that what translates as a planctomycetal LGT today might turn out to be of chlamydial origin upon further exploration of the extant Chlamydiales diversity. On the other hand, a bona fide planctomycete gene could very well have replaced a plastidial gene of a different phylogenetic origin. In any case, the presence of this foreign gene testifies that the tight cyanobacterial control of the metabolic flux to tryptophan has been lost very early on after plastid endosymbiosis.

We believe that the high proportion of chlamydial LGTs to tryptophan metabolism in Archaeplastida is not coincidental. What would be the purpose for Chlamydiales to manipulate cyanobacterial tryptophan metabolism? We surmise that Chlamydiales deregulated and increased the flux of cyanobacterial tryptophan synthesis at a very early stage of plastid endosymbiosis, for two non-mutually exclusive reasons. The first would be to supply more tryptophan for the synthesis of the Trp-rich UhpC and ATP import proteins. This explanation depends on the amount of transporter protein required to trigger symbiosis. Nevertheless, extant plastidial carbohydrate transporters are among the most abundant proteins on the plastid's inner membrane (Joyard et al., 1982). The second reason would be to provide an ample supply of the previously scarce and very highly treasured tryptophan for the chlamydial partner of the “Ménage à trois.” The overflow of tryptophan in the inclusion could also be further exported to the cytosol, for the host's benefits. Export of tryptophan from the cyanobiont to the inclusion requires a transporter, which is apparently not encoded by single-cell cyanobacteria (Zhao et al., 1994; this work). The phylogeny presented in Figure 2B demonstrates that the TyrP/Mtr *E. coli* transporters (Sarsero et al., 1991) known to import respectively tyrosine and tryptophan in Chlamydiaceae (Bonner et al., 2014) probably defines yet an unexpected additional LGT from Chlamydiales to Archaeplastida. This LGT possibly escaped previous detection because of the proximity of a subset of candidate donor cyanobacterial sequences (Figure 2B). Indeed, only one previous report (Becker et al., 2008) considers this gene as a candidate LGT. While the very few deltaproteobacteria found within the Archaeplastida do not qualify as candidate donors, the cyanobacteria concerned consist almost exclusively of Nostocales. Only two Chroococcales sequences were detected, the most basal being Acaryochloris which diversified after plastid endosymbiosis (Shih et al., 2013). This clade distribution is consistent with an LGT from an Archaeplastida ancestor to an Acaryochloris-like clade. The phylogeny does not discriminate between two alternatives. On the one hand, the gene might have originated in Nostocales, then was transferred to Archaeplastida, Chlamydiales and finally the full suite of γ-proteobacteria. On the other hand, the latter might have been of ancient proteobacterial origin with a transfer to Archaeplastida through Chlamydiales and the final capture of the gene by Nostocales. We favor a non-cyanobacterial origin for TyrP among the deeply rooted γ-proteobacteria as the latter predate by far Nostocales diversification (Battistuzzi et al., 2004).

## Conclusions and future directions

In this perspective, we propose that a Trp operon from a Chlamydiales was donated to the cyanobacterial ancestor of plastids through conjugation within a common chlamydial inclusion. Conjugation and direct transfer of genes in the cyanobiont genome would have offered a unique opportunity for immediate expression of chlamydial proteins at the earliest stages of symbiosis when protein translocation to the evolving plastid was not yet established.

Increasing the flux to tryptophan after transfer of the chlamydial operon was mostly beneficial to both the pathogen and its host in face of restricted tryptophan supply. In addition, the synthesis of large amounts of high Trp containing carbon transporters responsible for the onset of plastid endosymbiosis may also have required an increase in Trp synthesis. This proposal is in line with the previously observed transfer of the *Simkania negevensis* Trp operon to *Coxiella burnetii* that probably happened by a similar mechanism in a common intracellular environment. It is also in line with the presence of a functional type IV conjugation machinery in both Simkaniaceae and Parachlamydiaceae which are the closest Chlamydiales at the root of the Archaeplastida in many documented LGTs.

It is probable that the insights gained and the logics applied here by looking at tryptophan metabolism can be extended to other plant pathways targeted by Chlamydiales LGTs. We indeed believe that detailed knowledge of Chlamydiales biology will lead to a deeper understanding of metabolic integration of plant plastid biochemical pathways. In this respect, whether extant archaeplastidal TyrP sequences encode bona fide plastidial tryptophan transporters remains a hypothesis worthy of future exploration.

## Author contributions

SB and UC coordinated the writing and wrote this manuscript together with inputs from all other listed co-authors. UC computed all phylogenetic trees. CC, MD, and DK made respectively the literature searches on Nostocales, intracellular gammaproteobacteria, and tryptophan transport.

## Funding

SB was supported by the CNRS, the Université de Lille CNRS, and the ANR grants “Expendo” (ANR-14-CE11-0024) and “ménage à trois” (ANR- 12-BSV2-0009).

### Conflict of interest statement

The authors declare that the research was conducted in the absence of any commercial or financial relationships that could be construed as a potential conflict of interest.
