# Supplementary material for: Was the Chlamydial Adaptative Strategy to Tryptophan Starvation an Early Determinant of Plastid Endosymbiosis?
Source: Front Cell Infect Microbiol. 2016 Jun 22;6:67. doi: 10.3389/fcimb.2016.00067 (PMC4916741; doi:10.3389/fcimb.2016.00067)
Supplement: Supplementary file 1 [file Presentation1.pdf]

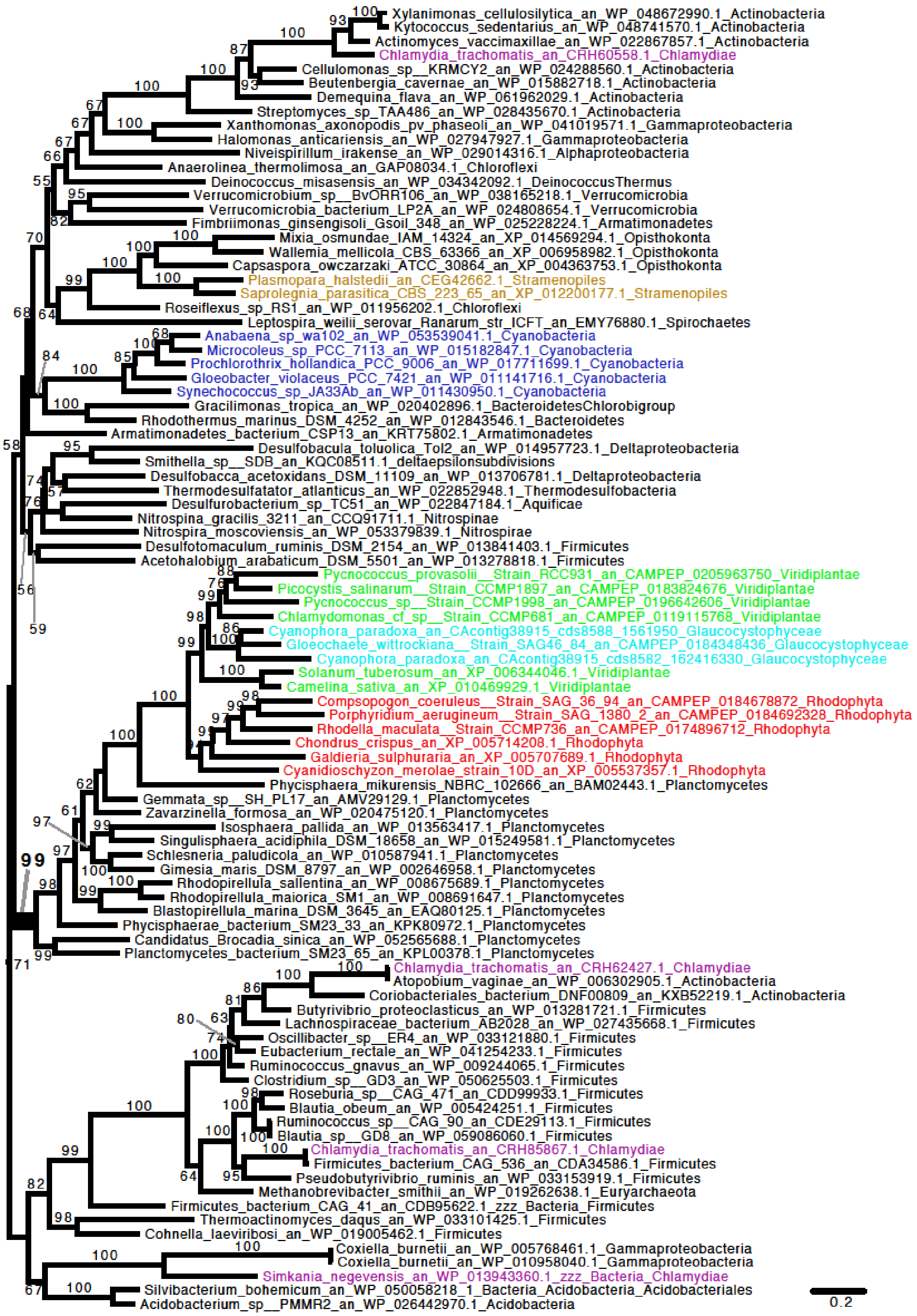

Supplementary Figure 1: Maximum-Likelihood (ML) phylogenetic tree of anthranilate synthase alpha subunit (TrpE). The tree was obtained with IQ-TREE (Nguyen et al., 2015) under the LG4X (Le et al., 2012) model with 1000 ultrafast bootstrap replicates (Minh et al.,

2013). Groups of interest are highlighted in purple (Chlamydiales), green (green algae and plants), red (red algae), cyan (Glaucophyta), and blue (Cyanobacteria). Lineages putatively derived from secondary endosymbiosis of Archaeplastida are displayed in brown. Ultrafast bootstrap values (UFBS) higher than 50 are indicated onto the branches. Cyanobacteria were reduced for convenience of display but contained the full diversity of accessible cyanobacterial sequences. No cyanobacterial sequence were found out of this cluster. The scale bars indicate the inferred number of amino acid substitutions per site. The trees were manually rooted for convenience of display. The nodes uniting Archaeplastida and their derived lineages to Chlamydiales by LGT are highlighted in bold. The anthranilate synthase alpha subunit tree shows a group with robust support (UFBS = 99) composed of Planctomycetes and all Archaeplastida.

References:

Le, S.Q., Dang, C.C., and Gascuel, O. (2012). Modeling Protein Evolution with Several Amino Acid Replacement Matrices Depending on Site Rates. *Mol. Biol. Evol.* 29, 2921–2936. doi:10.1093/molbev/mss112.

Minh, B.Q., Nguyen, M.A.T., and von Haeseler, A. (2013). Ultrafast Approximation for Phylogenetic Bootstrap. *Mol. Biol. Evol*. 30, 1188–1195. doi:10.1093/molbev/mst024.

Nguyen, L.-T., Schmidt, H.A., von Haeseler, A., and Minh, B.Q. (2015). IQ-TREE: A Fast and Effective Stochastic Algorithm for Estimating Maximum-Likelihood Phylogenies. *Mol. Biol. Evol*. 32, 268–274. doi:10.1093/molbev/msu300.
